# The Effect of Resilience and Family Support Match on Psychological Distress among Women in the Menopausal Transition Based on Polynomial Regression and Response Surface Analysis

**DOI:** 10.3390/ijerph192114165

**Published:** 2022-10-29

**Authors:** Qing Wang, Di Zhao, Miao Zhou, Xiangyu Zhao, Yiming Gao, Junyan Duan, Cong Cao, Ping Li

**Affiliations:** Department of Health Psychology, School of Nursing and Rehabilitation, Shandong University, Jinan 250012, China

**Keywords:** resilience, family support, psychological distress, polynomial regression, response surface analysis, menopausal transition

## Abstract

Menopausal transition (MT) is a natural process in women from reproductive decline to aging. During this period, women present with an increased prevalence of psychological distress. The aim of this study was to investigate how resilience and family support interact to influence psychological distress among women during MT. A convenience sampling method was used to recruit 858 women during MT from Shandong Province. All participants completed the 10-item Connor–Davidson Resilience Scale, the family care degree questionnaire, and the Kessler Psychological Distress Scale. Data were analyzed by using SPSS 24.0. Response surface analysis was used for polynomial regression and response surface analysis. The results of response surface analysis show that in the case of agreement between resilience and family support, the joint effect manifested as a negative curve (inverted U shape) related to the psychological distress of women during MT. In the case of disagreement, the joint effect manifested as a negative curve (inverted U shaped) related to psychological distress. Specifically, with increased variance in the degree of resilience and family support, women experienced less psychological distress. Both resilience and family support play an important role in protecting women from psychological distress, either alone or in combination. Future intervention studies targeting women during MT should consider the coordinated actions of resilience and family support.

## 1. Introduction

Menopause transition (MT) refers to the period of transition from reproductive (premenopause) to non-reproductive (postmenopause) life in women, representing a vulnerable period for women [1,2]. During this period, owing to fluctuations in estradiol and testosterone, in addition to vasomotor symptoms (hot flashes and night sweats), women can also experience a cascade of psychological distress symptoms (anxiety, depression, and irritability) [3]. Furthermore, during MT, women tend to be middle-aged, often playing a multifaceted role as wife, mother, caregiver to elderly parents, and employee, making them prone to experiencing psychological distress. These stacking psychological distresses caused by internal and external factors could result in a significant reduction in quality of life in women during MT [4], which deserves the attention of researchers and clinicians.

Resilience, as an internal protective factor, is defined as the capacity to successfully adapt in the face of adversity or risk, which plays a role in chronic stress responses [5,6,7]. According to the stress coping theory [8], individuals with high resilience can successfully adapt to changing situations, effectively regulate emotional reactions, and build mutually supportive relationships with others, thereby alleviating psychological distress [9,10]. Given that MT serves as a chronic and lasting stressor, during this period, women are generally affected by long-term hormonal and external pressures, and psychological distress can persist [11,12]. Little is known about the relationship between resilience and psychological distress in women during MT, and further research is warranted.

Social support, as a key external resource and buffer system, plays a significant role in the face of stress or adversity [13,14]. In essence, social support is a multidimensional concept, including support from family, friends, and others [15]. In the context of Chinese traditional culture, family is usually regarded as the closest social support system for women [16]. Individuals with high levels of family support feel more secure, are easily aware of the positive aspects of event, and seek coping strategies, reducing psychological stress [17,18]. During MT, women are in a vulnerable “sandwich” period, with elderly family members to support and children to nurture, suffering from long-term psychological distress [19]. Considering the importance of family support, it makes sense to explore its impact on psychological distress in women.

As mentioned above, both resilience and family support play a role in reducing psychological distress. However, individuals with high resilience do not necessarily have high levels of family support and vice versa. Previous literature has suggested a low to moderate correlation between resilience and family support [20,21], with four possible combinations of these factors: equally high resilience and family support, equally low resilience and family support, high resilience combined with low family support, and high family support combined with low resilience. In general, individuals invoke internal and external resources simultaneously when faced with stress. Hence, it important to examine how the various combination of resilience and family support affect psychological distress in women during MT. Polynomial regression and response surface methods have been broadly applied to test how the combinations of two predictor variables correlate with an outcome variable, particularly with respect to consistency and non-consistency measures [22]. A response surface map can be used to directly reflect complex three-dimensional relationships to provide insights and comprehensive information about the investigated effect [23].

Accordingly, in the present study, we examined the research questions unilateral and bilateral perspectives, with the aim of investigating how resilience and family support interact to influence psychological distress in women during MT. This is the first study to focus on the impact of the coordinated role of internal and external resources on psychological distress; the reported results can help to optimize the use of resources and improve the mental health of women during MT.

## 2. Materials and Methods

### 2.1. Participants and Procedure

A cross-sectional study was conducted from December 2017 to August 2018, including 858 women in Dezhou city, Shandong Province, China. The inclusion criteria included age 40 to 60 and no current psychiatric disease or cognitive dysfunction. Women were excluded from the study if they had undergone a hysterectomy or ovariectomy or had other conditions that interfered with the normal functionality of sex hormones. Participants were recruited by posting flyers throughout communities in Dezhou city. After the acquisition of informed consent, the participants were asked to complete questionnaires regarding sociodemographic characteristic, resilience, family support, and psychological distress. The study was approved by the Research Ethics Committee of the affiliated institution.

### 2.2. Measures

#### 2.2.1. Sociodemographic Questionnaire

A researcher-designed questionnaire was used to assess sociodemographic and clinical variables, primarily age, body mass index, marital status, economic status, chronic diseases, and gynecological diseases (including gynecological inflammation, myomectomy, and other diseases).

#### 2.2.2. Connor–Davidson Resilience Scale (CD-RISC)

A simplified Chinese version of the 10-item Connor–Davidson Resilience Scale (CD-RISC-10) was adopted to measure resilience [24,25]. The Chinese version of the inventory is widely used in the Chinese population and shows excellent validity. Each item was scored according to a five-point scale, from zero (never) to four (always) points. Higher scores represent better resilience. The Cronbach’s alpha coefficient in this study was 0.870.

#### 2.2.3. Family Care Degree Questionnaire (APGAR)

Family support was measured by the family care degree questionnaire (APGAR), which included five items: fitness, collaboration, growth, feeling, and intimacy [26]. Each of these items was evaluated on a three-point scale, with scores ranging from zero (hardly ever) to two (nearly always). Sum scores were calculated and ranged from zero to ten. In accordance with the standard classification, an overall score of 1 to 3 indicates severe family dysfunction, an overall score of 4 to 6 indicates moderate family functioning, and an overall score of 7 to 9 indicates good family functioning. The higher the score, the better the family functioning and the higher the degree of family care perceived by individuals. The Cronbach’s alpha coefficient in the present study was 0.771.

#### 2.2.4. Kessler Psychological Distress Scale (K10)

The level of psychological distress was assessed using the Kessler Psychological Distress Scale (K10) [27]. A total of ten items were scored on a five-point scale of one (almost none) to five (all the time) for each item, with a total score of ten to fifty. Based on the total score, psychological distress was classified into three levels: mild psychological distress (10–24 points), moderate psychological distress (25–29 points), and serious psychological distress (30–50 points). The K10 commonly used worldwide to assess psychological distress in various populations. In the current study, the Cronbach’s alpha coefficient was 0.874.

### 2.3. Data Analysis

SPSS version 24.0 statistical software (IBM Corp., Armonk, NY, USA) was used for all analyses. Correlations and descriptive statistics were calculated first. Continuous variables, such as body mass index and age, were represented by mean (M) and standard deviation (SD), whereas categorical variables were characterized by frequency (N) and percentage (%). Then, the consistency and inconsistency of resilience and family support and their relationship with psychological distress were analyzed using discrepancy analysis, polynomial regression, and response surface analysis.

First, in order to identify agreement or disagreement between resilience and family support, it was necessary to explore the basic rate of discrepancies between the two variables and identify the distribution of differences in the sample. According to the recommendations of Shanock et al., this should be done prior to conducting polynomial regression to provide a theoretical basis for further exploration of the discrepancies [28]. Then, the percentages of congruence and incongruence were examined. The existence of differences between resilience and family support (i.e., more than half of the sample) provided a theoretical basis for further polynomial regression analysis.

A polynomial regression was then performed in SPSS software to calculate the surface values. According to the procedure proposed by Atwater et al. [29], the predictors (resilience and family support) were first centralized, which helped to interpret and reduce the potential for multicollinearity. Next, three new variables were constructed, including the square of the centered resilience variable, the cross product of the centered resilience variable and the family support variable, and the square of the centered family support variable. Then, polynomial regression analysis was performed by regressing the outcome variable (psychological distress) on the centered predictor variables (resilience and family support), the product of centered resilience and family support, the centered resilience squared, and the centered family support squared terms into the regression equation. If R^2^ (the variance of the outcome variable explained by the regression equation) was significant and not equal to zero, the unstandardized beta coefficients of this analysis were then applied to compute the response surface patterns, which were used to generate three-dimensional graphs. Four surface test values were examined: a_1_, a_2_, a_3_, and a_4_. a_1_ represents the slope of the perfectly congruent line associated with psychological distress. The curvature of the perfectly congruent line associated with psychological distress was assessed according to a_2_. If there was a non-linear relationship along the perfectly congruent line with respect to psychological distress, a_2_ would be significant but a_1_ would not. The curvature of the line of inconsistencies associated with psychological distress was assessed by calculating a_4_, indicating the degree of difference between resilience, family support, and outcomes. The slope of the line of inconsistencies associated with psychological distress was assessed by calculating a_3_, indicating the direction of the difference.

## 3. Results

### 3.1. Descriptive Statistics

Differences of sociodemographic and clinical characteristics of the sample of 858 during MT in terms of psychological distress are provided in Table 1. The mean age of the women in this study was 49.83 ± 5.01 years old. Among all respondents, more than 50% of the women felt they balanced their income and expenses. Most women reported no chronic diseases or gynecological diseases, accounting for 58.28% and 75.41% of the sample, respectively. Statistically significant differences were observed in terms of psychological distress discrepancies based on economic conditions, chronic diseases, and gynecological diseases (Table 1).

### 3.2. Correlation Analysis between the Variables

The correlations between the examined variables are presented in Table 2. All analysis results are statistically significant at the level of *p* < 0.01 (two-tailed). Resilience (*r* = −0.230, *p* < 0.01) and family support (*r* = −0.208, *p* < 0.01) exhibit a minimal negative correlation with psychological distress.

### 3.3. Discrepancy Analysis

Table 3 shows the frequencies of resilience scores over, under, and in agreement with family support scores. Specifically, 33.68% of the women reported higher levels of resilience than levels of family support, and compared to the level of family support, 30.07% of women scored comparatively similar in terms of resilience (i.e., the difference between the standardized scores was less than half a standard deviation); nevertheless, 36.25% of the women reported lower levels of resilience than family support. The descriptive information indicates that a considerable number of observations differ, and it made practical sense to further examine how agreement and disagreement in resilience and family support were related to psychological distress.

### 3.4. Polynomial Regression and Response Surface Analyses

The results of the polynomial regression with resilience and family support of women during MT, along with the *X*_1_ ^2^, *X*_2_ ^2^, and *X*_1_*X*_2_ terms as predictors of psychological distress, are shown in Table 4. The significant *R*^2^ value of 0.132, *p* < 0.01, suggested that it was appropriate to proceed with the response surface analysis and that roughly 13.2% of the variance in psychological distress could be explained by the variables in this equation.

**Figure 1:**
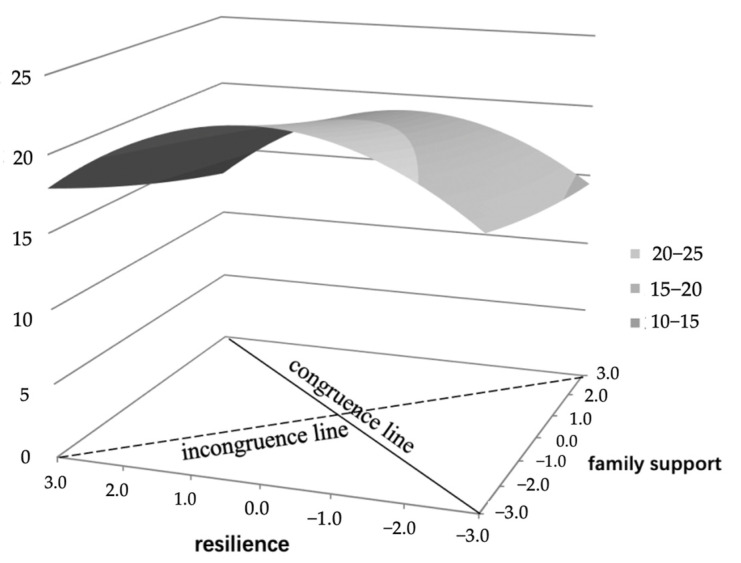
Response surface graphs showing psychological distress predicted by resilience and family support.

A three-dimensional response surface graph was generated using the coefficients obtained from the regression analysis to provide a better understanding of the relationship between resilience, family support, and psychological distress by testing the four surface values (Table 5, Figure 1). The significantly negative curvature of the congruence line (a_2_) indicates a concave surface, implying that equally high or low resilience and family support were associated with lower psychological distress compared to equal but midrange resilience and family support. The curve of the incongruence line (a_4_) is significantly negative, illustrating that psychological stress decreased sharply as the degree of discrepancy between resilience and family support increased. Our results demonstrate that the slope of the perfectly congruent line (a_1_) and the slope of the disagreement line (a_3_) were not statistically significant.

## 4. Discussion

The goal of the current study was to assess the effect of resilience and family support match on psychological distress among women during MT using polynomial regression and response surface analysis; to the best of our knowledge, this is the first such study to be published in the literature. In the case of congruence between resilience and family support, the joint effect was curvilinearly related to psychological distress in women during MT. In the case of incongruence between resilience and family support, a complementary effect was found, meaning that the greater the difference, the less psychological distress experienced by women.

In our study, we observed significant non-linear resilience–family support convergence effects. We noted that equally high resilience and family support was associated with reduced psychological distress, suggesting that the combined effect could be effective in reducing psychological distress among women during MT. For women, MT is a time of stress exposure [30]. During this period, living with chronic fatigue and painful symptoms (such as fibromyalgia) over time could wear down women’s cognitive–emotional reserve, leading to forms of despair and withdrawal from usual activities, resulting in increased psychological distress [30,31]. However, women with high resilience during this period have sufficient cognitive skills and emotional management ability and are more likely to proactively adapt to stress, which is consistent with the findings of previous studies [32,33]. Meanwhile, higher levels of family support related to lower psychological distress; similar findings have been reported in other studies [16]. A possible reason for this result could be that under the influence of Chinese traditional culture, family is the core of women’s life, meaning that they obtain support primarily from family [16]. Support from family can help women maintain positive emotions, such as vitality, hope, and optimism, during MT, which are conducive to expanding the influence of positive emotions, promoting coping abilities, and thus alleviating psychological distress [34].Therefore, the combination of high resilience and high family support is conducive to alleviating psychological distress in women during MT. Interestingly, low resilience combined low family support was associated with lower psychological distress compared to equal but midrange resilience and family support. According to stress theory, when individuals perceive low levels of stress, they usually do not mobilize their internal and external resources to cope and do not experience high levels of psychological distress [35]. Therefore, for some women during the MT who did not perceive high levels of stress, high levels of resilience and family support would not be stimulated, resulting in low levels of psychological distress.

In the case of incongruence between resilience and family support, a complementary relationship was also observed. In particular, either high resilience combined with low family support or low resilience combined with high family support was correlated with low psychological distress, indicating that both internal and external resources play an important role in reducing psychological distress in women during MT. On the one hand, women with high levels of resilience had enough inner strength to resist external stress, as well as to compensate for the lack of family support, therefore experiencing less psychological distress. On the other hand, low levels of resilience in women during MT can be offset by strong support from the family, thus alleviating psychological distress.

Although contributing important data, the present study is subject to several limitations. First, we used cross-sectional data; therefore, care should be taken making causal judgements based on the reported results. Future studies should collect longitudinal data to confirm the potential causal relationships reported herein. Second, we a self-reported measurement method to determine the stages of MT based on recall of the menstrual cycle. Consequently, the accuracy of the results may be influenced by recall bias. Finally, we did not take some confounding factors (such as the use of birth control) into consideration.

## 5. Conclusions

The present study is the first to explore how internal (resilience) and external (family support) resources interact to influence psychological distress in women during MT using polynomial regression and response surface analysis. Herein, we provided a novel visual representation to improved understanding of the complex relationship between resilience, family support, and psychological distress. Our findings suggest that the joint effect of resilience and family support can influence psychological distress in women during MT. In particular, when resilience and family support were in congruence, the joint effect on psychological distress in women during MT had a negative curvilinear relationship; when resilience and family support were in incongruence, psychological distress increased with decreased discrepancy between resilience and family support. Our findings suggest that both resilience and family support play an important role in protecting women from psychological distress, either alone or in combination. Our findings provide a basis for future intervention studies targeting women during MT, which should consider the coordinated actions of resilience and family support.

## Figures and Tables

**Table 1:** Differences in sociodemographic and clinical characteristics of 858 women during MT in terms of psychological distress (*n* = 858).

Variable	N (%)	Psychological Distress	t/F	*p*
BMI (kg/m^2^)			0.082	0.921
thin or normal weight	104 (12.12%)	18.53 ± 6.34		
overweight	373 (43.47%)	18.71 ± 6.11		
obese	374 (43.59%)	18.56 ± 5.73		
Education level			0.177	0.838
illiterate	106 (12.35%)	18.75 ± 6.68		
primary or junior school	701 (81.71%)	18.62 ± 6.06		
polytechnic or high school	51 (5.94%)	18.14 ± 6.46		
Marital status			0.175	0.861
married	843 (98.25%)	18.61 ± 6.14		
other	15 (1.75%)	18.33 ± 7.24		
Economic conditions			3.186	0.042
insufficient	202 (23.54%)	19.52 ± 6.30		
sufficient for essentials	552 (64.34%)	18.41 ± 6.10		
more than sufficient	104 (12.12%)	17.91 ± 6.06		
Chronic diseases			−4.888	<0.001
no	500 (58.28%)	17.76 ± 6.05		
yes	358 (41.72%)	19.82 ± 6.11		
Gynecological diseases			−4.404	<0.001
no	647 (75.41%)	18.09 ± 5.68		
yes	211 (24.59%)	20.21 ± 7.21		

**Table 2:** Correlation analysis of psychological variables in 858 women during menopausal transition.

Variable	M	SD	1	2	3
1. Resilience	24.71	7.25	1		
2. Family support	7.16	2.49	0.190 **	1	
3. Psychological distress	18.61	6.16	−0.230 **	−0.208 **	1

Note: ** *p* < 0.01.

**Table 3:** Frequencies of resilience levels over, under, and in agreement with family support levels.

Agreement Group	N	Percentage	Mean Resilience	Mean Family Support
Resilience higher than family support	289	33.68	1.47	0.22
In agreement	258	30.07	0.72	1.39
Resilience lower than family support	311	36.25	−0.02	2.22

**Table 4:** Polynomial regression results for the effects of resilience and family support.

Variable	Psychological Distress		
	Model 1	Model 2	Model 3
Intercept	13.726 **	19.549 **	15.139 **
Control Variables			
Economic conditions	−0.619	−0.356	−0.313 **
Chronic diseases	1.723 **	1.731 **	1.666 *
Gynecological diseases	1.752 **	1.552 **	1.590 *
Predictors			
Resilience		−0.121 **	−0.072 **
Family support		−0.421 **	−0.654 *
Resilience × resilience			−0.569 *
Resilience × family support			−0.050 **
Family support * family support			0.034 **
*R* ^2^	0.054 **	0.109 **	0.132 **
*F*	12.054 **	17.270 **	14.346 **
Δ*R*^2^		0.055 **	0.023 **
Δ*F*		26.272 **	7.686 **

Note: * *p* < 0.05, ** *p* < 0.01.

**Table 5:** Response surface parameters of resilience and family support in the prediction of psychological distress.

Response Surface Parameter	Psychological Distress	
	β	CI
Congruence (X_1_ = X_2_) line		
a_1_ (slope)	−0.727	(−1.587~0.132)
a_2_ (curvature)	−0.585 **	(−0.924~−0.200)
Incongruence (X_1_ = X_2_) line		
a_3_ (slope)	0.582	(−0.185~1.325)
a_4_(curvature)	−0.484 *	(−0.884 ~ −0.072)

Note: X_1_: resilience, X_2_: family support; * *p* < 0.05, ** *p* < 0.01.

## Data Availability

The data presented in this study are available upon request from the corresponding author.
